# The ORF45 Protein of Kaposi’s Sarcoma-Associated Herpesvirus and Its Critical Role in the Viral Life Cycle

**DOI:** 10.3390/v14092010

**Published:** 2022-09-11

**Authors:** Natalie Atyeo, Bernadett Papp

**Affiliations:** 1 Department of Oral Biology, University of Florida College of Dentistry, Gainesville, FL 32610, USA; 2 UF Genetics Institute, University of Florida, Gainesville, FL 32610, USA; 3 UF Health Cancer Center, University of Florida, Gainesville, FL 32610, USA; 4 UF Informatics Institute, University of Florida, Gainesville, FL 32610, USA; 5 UF Center for Orphaned Autoimmune Disorders, University of Florida, Gainesville, FL 32610, USA

**Keywords:** herpesvirus, tegument, KSHV, gammaherpesvirus, immediate-early gene, ORF45, RSK, MAP kinase pathway, interferon

## Abstract

Kaposi’s sarcoma-associated herpesvirus (KSHV) protein ORF45 is a virion-associated tegument protein that is unique to the gammaherpesvirus family. Generation of KSHV ORF45-knockout mutants and their subsequent functional analyses have permitted a better understanding of ORF45 and its context-specific and vital role in the KSHV lytic cycle. ORF45 is a multifaceted protein that promotes infection at both the early and late phases of the viral life cycle. As an immediate-early protein, ORF45 is expressed within hours of KSHV lytic reactivation and plays an essential role in promoting the lytic cycle, using multiple mechanisms, including inhibition of the host interferon response. As a tegument protein, ORF45 is necessary for the proper targeting of the viral capsid for envelopment and release, affecting the late stage of the viral life cycle. A growing list of ORF45 interaction partners have been identified, with one of the most well-characterized being the association of ORF45 with the host extracellular-regulated kinase (ERK) p90 ribosomal s6 kinase (RSK) signaling cascade. In this review, we describe ORF45 expression kinetics, as well as the host and viral interaction partners of ORF45 and the significance of these interactions in KSHV biology. Finally, we discuss the role of ORF45 homologs in gammaherpesvirus infections.

## 1. Introduction

Kaposi’s sarcoma-associated herpesvirus (KSHV) is a double-stranded DNA gammaherpesvirus that is the etiologic agent of Kaposi’s sarcoma [1], primary effusion lymphoma [2] and multicentric Castleman’s disease [3]. KSHV has a broad tropism in adherent cell lines, where the default pathway following primary infection is the establishment of viral latency [4]. However, lytic viral infections have been detected in select cell types, including primary human tonsillar B cells, oral epithelial cells and lymphatic endothelial cells [5,6,7,8,9]. Importantly, although the majority of KSHV-induced tumors are composed of latently infected cells, spontaneous lytic reactivation can occur in a subset of cancer cells, which is posited to be important for sustained tumorigenesis [10]. Lytic reactivation results in a cascade-like expression of viral immediate-early (IE), early (E) and late (L) genes in a highly regulated temporal manner. KSHV encodes over eighty genes, many of which have roles in host immune evasion, that are crucial for facilitating lytic viral replication and virus production [11,12]. One of the earliest recognized factors involved in suppression of the host immune response against KSHV is ORF45, which was elegantly demonstrated in 2002 by Zhu et al. [13]. As both a part of the KSHV virion and an immediate-early gene, ORF45 plays distinct roles during several phases of the viral life cycle. Given the integral role of ORF45 during KSHV infection and reactivation, a full understanding of its various functions is essential to assess how ORF45 and/or ORF45-regulated pathways could be targeted for antiviral therapies. The objective of this review is to summarize the many different functions of the multifunctional KSHV-encoded ORF45 protein and its gammaherpesvirus homologues.

## 2. Expression of ORF45 as an Immediate-Early Gene

Like other herpesviruses, KSHV encodes four classes of genes: immediate-early, early, late and latent genes. While the lytic program is suppressed during latency, upon reactivation, the full cascade of immediate-early, early and late genes is expressed in a regulated temporal manner. Immediate-early genes are expressed in the absence of prior viral protein expression, and therefore, they are defined as genes whose expression is resistant to protein synthesis inhibitor treatment following chemically induced reactivation [14]. As the first genes expressed in the first hours of lytic reactivation, immediate-early genes play key roles in the viral life cycle and host immune evasion [15]. Several immediate-early genes of KSHV were identified following reactivation of latently infected Primary Effusion Lymphoma cells, including the lytic switch protein ORF50, or Replication and Transcription Activator (RTA) and ORF45 [16]. Recent genome-wide approaches also identified ORF45 to be induced within 8 h of induction in the iSLKBAC16 KSHV^+^ epithelial cell line and within 4 h of induction in KSHV^+^ B cell lymphoma cell line BCBL1 [16,17], reinforcing the notion that ORF45 is expressed as an immediate-early gene in the KSHV lytic cycle. In addition, transcriptomic analysis following de novo infection of peripheral blood mononuclear cells (PBMCs), CD14+ cells and telomerase immortalized vascular endothelial (TIVE) cells, revealed relative abundance of actively transcribed ORF45 mRNA at 4 h post-infection, indicating its rapid accumulation in host cells following KSHV infection [18]. As detailed below, the widely expressed lytic viral ORF45 plays a crucial role both during de novo infection and lytic reactivation.

## 3. ORF45 Structure and Localization

KSHV ORF45 is a 1.7-kb gene, which encodes the 407-amino acid ORF45 protein. The ORF45 gene is part of the orf47-orf46-orf45 gene cluster, and ORF45 is expressed from this tricistronic mRNA [19,20,21]. In addition to expressing the full ORF45 protein, the alternatively spliced mRNA also yields two gene products, ORF47/45-A and ORF47/45-B [22]. The ORF45 protein has been shown to localize to both the nucleus and the cytoplasm, with the ability to shuttle between the two compartments [23]. While exogenously expressed ORF45 is predominantly located in the cytoplasm in HeLa [24] and 293T [13] cells, ORF45 was also located in nuclear replication compartments in reactivated B-cells [25]. ORF45 possesses a nuclear localization sequence (NLS) from amino acids 297–300 and a nuclear export sequence (NES) from amino acids 284–294 [23]. NLS-defective KSHV mutants, but not NES-defective KSHV mutants, had a decreased production of viral progeny, demonstrating that ORF45 subcellular localization is linked to its pro-viral role [23]. Interestingly, ORF45 has an acidic domain between amino acids 90 and 115 that is characteristic of nuclear proteins and transcriptional activators [16] suggesting that ORF45 could also function as a transcription regulatory factor, but its potential function in a chromatin environment is still largely unknown.

## 4. ORF45 as a Component of the Tegument of KSHV

Proteomic analysis of KSHV virions revealed that ORF45 is also part of the viral tegument [26,27]. The virion-associated tegument of herpesviruses is located between the viral nucleocapsid and envelope, and its components have functional roles in virion entry, assembly, egress and modulation of host signaling pathways [28,29,30]. ORF45 was among 24 KSHV proteins identified via a mass spectrometry analysis of purified virions isolated from B-cell lymphoma cells [27]. Moreover, ORF45 was among the proteins that were resistant to trypsin digestion of purified virions only in the absence of detergent, a classical indication that it is a tegument protein [26,27]. Recent cryo-electron microscopy studies have allowed visualization of the gammaherpesvirus tegument as a structured organization of proteins which interact within the tegument layer and with capsid and envelope proteins [31]. More recently, the composition of the KSHV virion was revisited with ultra-high resolution Qq time-of-flight mass spectrometry, and ORF45 was confirmed as a virion-associated protein using this method [32]. Finally, in addition to being detectable within infectious virions, ORF45, along with several other tegument proteins, are also found at comparable levels in KSHV virus-like vesicles (VLVs), which are produced following productive lytic infection of cells in high number but lack the viral capsid and genome [33]. While VLVs have been shown to modulate host differentiation signaling to promote infection, the contribution of the individual viral factors in VLVs remains to be identified. In sum, ORF45 is one of the earliest KSHV lytic factors present in the host cell following both de novo infection, when it is directly delivered into the host cell as a tegument protein, and also within hours of lytic reactivation as an immediate-early protein.

## 5. ORF45 Interactions with Viral Proteins

**Figure 1:**
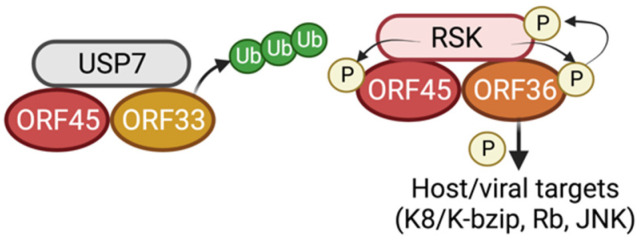
ORF45 interaction with viral proteins. (Left) ORF45 binds to viral tegument protein ORF33 via its C-terminus and stabilizes ORF33 via interaction with host de-ubiquitinase USP7, which prevents the ubiquitylation and proteasomal degradation of ORF33 (Right) ORF45 binds to the KSHV serine/threonine kinase ORF36 in a complex with host p90 ribosomal s6 kinase (RSK), which phosphorylates both viral targets at its target RxRxxS*/T* motif. This interaction promotes subsequent phosphorylation of RSK by ORF36, as well as phosphorylation of ORF36 downstream targets (e.g., K8, Rb, JNK).

ORF45 interacts with several KSHV lytic proteins, and these interactions are functionally relevant for the KSHV life cycle (Figure 1). ORF45 was shown to associate with several capsid proteins and tegument proteins, in line with its key role as a structural and/or functional hub of tegument organization [34]. Through mass spectrometry analysis, ORF45 was shown to interact with and stabilize ORF33, a tegument protein which is conserved among herpesviruses and which plays a role in viral particle transport through cellular vesicles [35,36,37]. The stabilization of ORF33 also requires ORF45 binding to host ubiquitin-protease USP7. Disruption of the ORF45/ORF33 interaction through mutation of the ORF45 C-terminal ORF33-interacting residues led to a decrease in the ORF45 and ORF33 packaged into viral particles, as well as a decrease in production of virus particles [35,38]. Additionally, ORF45 is phosphorylated by the viral protein kinase ORF36, leading to an interaction between the two proteins that stabilizes ORF36. Importantly, ORF36-null mutants are deficient in primary infection, emphasizing the essential pro-viral role of ORF45 in shielding ORF36 from proteasomal degradation [39,40].

## 6. Role of ORF45 in the Viral Life Cycle

Given the kinetics of ORF45 expression, it is positioned to exert a crucial role in several phases of the KSHV life cycle. Multiple studies have employed mutagenesis of the ORF45 protein in order to further dissect these roles (Table 1).

**Figure 2:**
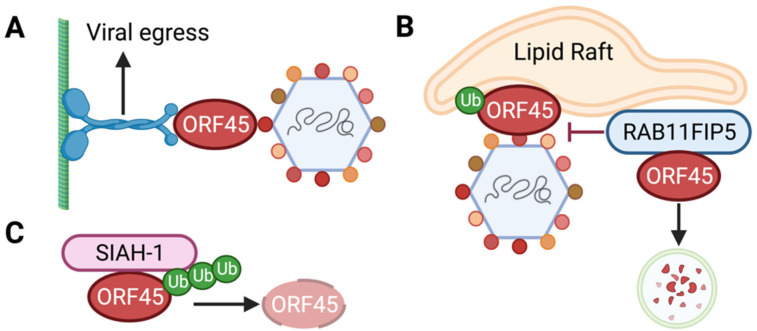
ORF45 role in the viral life cycle. (**A**) ORF45 associates with the cargo-binding domain of the KIF3A subunit of kinesin-2, which mediates the association of the viral capsid-tegument complex with microtubules, promoting viral egress. (**B**) ORF45, which is mono-ubiquitylated at lysine 297, mediates association of the viral capsid-tegument complex with lipid rafts targeted to the trans-Golgi network for eventual viral envelopment and egress. Host RAB11 family-interacting protein RAB11FIP5 interferes with ORF45 targeting to lipid rafts by interacting with ORF45 and promoting its lysosomal degradation, thereby inhibiting the endosomal trafficking of viral particles. (**C**) The SIAH-1 E3 ubiquitin ligase interacts with ORF45 leading to ORF45 ubiquitylation and degradation.

In addition, the engineering of mutant KSHV viruses via bacterial artificial chromosome (BAC)-based recombination enabled studies of ORF45’s role in the viral life cycle in the context of KSHV de novo infection and reactivation [47,48]. Originally, an ORF45-null virus was created using a KSHV BAC36 clone, and in this system ORF45 deletion did not have an effect on lytic viral gene expression following viral reactivation or de novo infection of 293T cells [49]. However, ORF45 deletion in the more recently engineered KSHV BAC16 clone, which is less prone to unintended homologous recombination, did affect a subset of late viral gene expression following reactivation of latently infected iSLK cells [42]. In both studies, the ORF45-deficient virus produced fewer progeny viruses, and the virions that were produced were less infectious than those produced by wild-type KSHV [42,49]. These findings suggest that ORF45 is required not only for effective primary infection, but also during the later stages of virus packaging and release from the host cell. Indeed, ORF45 has been shown to interact with several host factors implicated in viral egress. Following capsid packaging and tegumentation, ORF45 mediates the assembly of the capsid-tegument complex onto the cargo-binding KIF3A subunit of motor protein kinesin-2 [50]. Disruption of this interaction or disruption of microtubules inhibits the release of virion particles, highlighting the role of ORF45 in viral particle trafficking to the cell periphery and release from the host cell (Figure 2A). Further, ORF45 promotes virion release through its interaction with lipid rafts, which is critical for the release of infectious virions [45]. Importantly, mono-ubiquitylation of ORF45 is necessary for association with lipid rafts and the trans-Golgi network, a pre-requisite to final viral envelopment and release [45]. However, the association of ORF45 with lipid rafts can be disrupted by host RAB11 family-interacting protein 5 (RAB11FIP5), which targets ORF45 for lysosomal degradation via endosomal trafficking [51]. Overexpression of RAB11FIP5 inhibits the release of infectious virions, highlighting the role of ORF45 as a key mediator of viral egress [51] (Figure 2B). Finally, ORF45 was also shown to associate with host SIAH-family proteins through a yeast two-hybrid screen [52]. The SIAH family of E3 ubiquitin ligases possess an N-terminal RING domain to direct the degradation of host substrates [53,54]. Expression of SIAH-1 with ORF45 leads to the degradation of ORF45 [52] (Figure 2C). The regulation of the expression of the essential lytic cycle-promoting protein ORF45 by RAB11FIP5 and SIAH-1 therefore presents a key avenue for targeting ORF45 levels antiviral therapeutics.

## 7. ORF45-Mediated Sustained Activation of RSK

The most studied role of ORF45 to date is its interaction with the host mitogen-activated protein kinase (MAPK) pathway. The MAPK signaling cascade responds to external stimuli to induce internal responses, including cellular proliferation and survival, through sequential phosphorylation of downstream pathways, including the extracellular signal-regulated kinase (ERK) pathway. Both DNA and RNA viruses have been shown to hijack the MAPK-ERK signaling pathway to promote the viral life cycle (reviewed in [55]). KSHV was also demonstrated to rapidly activate the ERK pathway following infection, and inhibition of the ERK pathway blunts viral infection [56]. One of the downstream targets of the ERK pathway is the family of p90 ribosomal s6 kinases (RSK 1–4), which have diverse cellular functions, including the regulation of transcription, translation, cell survival and the cell cycle [57,58]. In a hallmark study by Dr. Fanxiu Zhu and colleagues, ORF45 was shown to interact with RSK1 and RSK2, leading to phosphorylation of both RSK1/RSK2 and ORF45 itself. Strikingly, activation of both RSK and ERK was diminished following primary infection or reactivation with ORF45-null virus [25]. A later proteomics study, which mapped the KSHV protein interactome using mass spectrometry analysis, also identified ORF45 as an interaction partner of three members of the RSK family and mitogen-activated protein kinases 1 and 3 (MAPK1, MAPK3) [59]. Interestingly, several pathogen proteins, including KSHV ORF45, Yersinia YopM protein and Theiler’s virus L protein, use a similar peptide motif to interact with RSKs, suggesting a process of convergent evolution of RSK-interacting proteins [60]. The mechanism by which ORF45 sustains activation of the ERK/RSK pathway is through the exploitation of kinase docking systems to bind to the RSK N-terminus. The binding of ORF45 to RSK stabilizes the interaction of ERK and RSK, creating a ternary complex that protects both proteins from dephosphorylation, maintaining them in an activated state [61,62]. Specifically, ORF45 uses a key phenylalanine residue at amino acid 66 to bind to RSK, and consequently, the exchange of this amino acid with alanine (F66A mutation) abolishes RSK binding [42]. Infection of cells with KSHV ORF45-F66A mutants leads to a reduced expression of late lytic genes, as well as a decrease in infectious virion production, highlighting the crucial role of ORF45-mediated RSK activation in the KSHV lytic cycle [42]. Consequently, the ORF45-RSK interaction has been explored as a therapeutic target. A short peptide, derived from ORF45 amino acids 56 to 76, which harbors the F66A mutation, has been shown to compete with ORF45 for RSK binding, inhibiting ORF45-driven RSK activation during lytic reactivation [63].

Several groups have explored the mechanism by which ORF45-mediated activation of RSK activates the KSHV lytic cycle. One of the known targets of RSK is eukaryotic translation initiation factor 4B (eIF4B), an important element of the translation initiation complex, which associates with eIF4A and eIF3 to promote ribosomal association with mRNA [64]. A screen of RSK targets revealed that ORF45 expression induced phosphorylation of eIF4B by RSK, enhancing the activity of host translational machinery, and subsequently contributing to the translation of KSHV lytic genes [65]. Recently, it has been shown that RSK1 targeting of eIF4B is dependent on RSK1 SUMOylation, a post-translational modification that affects RSK1 substrate specificity and its ability to promote the KSHV life cycle [66]. In fact, RSK1 SUMOylation is driven by ORF45, which acts as a SUMO E3 ligase through two SUMO-interacting motifs that are distinct from the domain required for RSK1 phosphorylation [44]. Importantly, the SUMO E3 ligase activity of ORF45 was shown to be critical in KSHV lytic replication [44,66].

In addition to its contribution to translational control, ORF45 also drives transcriptional activation of HIV-1 long-terminal repeats (LTRs) and can act synergistically with the HIV Tat protein [67]. ORF45 mediates this LTR activation through RSK2 signaling, which leads to increased RNA polymerase II occupancy in the HIV-1 LTRs [68]. Interestingly, expression of gammaherpesvirus ORF45 homologs failed to activate HIV LTRs, indicating a unique role for KSHV ORF45 in HIV reactivation upon KSHV infection. Moreover, ORF45-sustained activation of ERK/RSK can also activate expression of KSHV late viral genes by promoting c-Fos accumulation in KSHV-infected cells [69]. Sustained MAPK activation leads to phosphorylation of c-Fos, a part of the AP1 family of transcription factors, and drives expression of a subset of c-Fos-dependent lytic genes [69,70]. Finally, ORF45-mediated activation of RSK leads to inhibitory phosphorylation of tuberous sclerosis complex (TSC), an upstream inhibitor of mTORC1 signaling, a phenomenon that is observed in lymphatic endothelial cells but not in blood endothelial cells [8]. These findings are clinically relevant given the observation that treating patients with mTORC inhibitors can lead to the regression of KS lesions [71,72]. Given the broad implications of sustained MAPK activation, ORF45 plays an essential role in modulation of host response through its RSK-activating function to support productive KSHV infection.

## 8. Regulation of Cellular p53 Signaling

As a tegument protein with immediate access to the host cellular environment after infection, ORF45 not only plays an essential role in host immune evasion, but also in evading the host DNA damage response. ORF45 was recently identified among a high-throughput screen of viral protein interactions with p53 [73]. Specifically, ORF45 inhibits the activity of host antitumor protein p53, which has been previously characterized during the KSHV latent phase but has not been described during lytic reactivation [74,75,76,77]. Following activation by an external stimulus, such as a double-stranded DNA break, p53 is phosphorylated and released from its negative regulators, the E3-ubuitin ligases murine double minute 2 (MDM2) and murine double minute X (MDMX) and is then bound and stabilized by the ubiquitin specific protease 7 (USP7) to act as a transcription factor for downstream targets (reviewed in [78]). ORF45 uses two mechanisms to interfere with p53 signaling. First, ORF45 interacts with the p53 de-ubiquitinating enzyme USP7, which leads to increased p53 ubiquitination and degradation [43]. Second, ORF45 directly binds p53 and directs p53 localization to the cytoplasm, preventing a p53-mediated response in the nucleus [43]. While p53 mutations are rarely detected in KSHV malignancies, the dysregulation of p53 function by ORF45 could blunt host protective responses against cellular transformation.

## 9. Evasion of Host Defenses

The first described role of ORF45 during KSHV infection was its contribution to evasion of the host immune response through the inhibition of type I interferon (IFN-α/β) activity. Through a yeast two-hybrid screen, ORF45 was found to bind cellular interferon regulatory factor 7 (IRF7) [13]. IRF7 is one of the crucial transcription factors that is necessary for the induction of IFN-α expression, which requires the phosphorylation and nuclear translocation of IRF7 [79,80]. ORF45 was initially shown to physically associate with IRF7 and block virus-induced phosphorylation of IRF-7, a critical step in the type I interferon response [13]. Further, while infection with wild-type KSHV does not trigger a cellular antiviral state, infection with an ORF45-knockout KSHV does activate the host antiviral response, as indicated by lower susceptibility to other viral infections, as well as increased interferon-stimulated gene (ISG) expression [81]. Two models have been proposed to describe the mechanism of ORF45-mediated IRF7 inhibition. First, ORF45 interacts with a predicted auto-inhibitory domain of IRF7, keeping IRF7 in a closed conformation that hides key residues for IRF7 activity, including the DNA-binding domain and phosphorylation sites [82]. Second, ORF45 competes directly with IRF7 as an alternative substrate for IKKε and TBK1, the upstream kinases of IRF7, as ORF45 is efficiently phosphorylated by these kinases on Ser41 and Ser162 [41]. ORF45 mutants in which one or both serine residues were replaced with alanine (ORF45-S41A, ORF45-162A, ORF45-S41/162A) were not efficiently phosphorylated by IKKε and TBK1. While complementation with wild type ORF45 lead to a dose-dependent decrease in IRF7 reporter (IFN-α1) activation following reactivation of ORF45-deficient iSLK-BAC16-stop45 cells, complementation with the ORF45-S41/162A mutant had a lesser inhibitory effect, indicating the role of Ser41/Ser162 phosphorylation in ORF45 inhibition of IRF7 activity [41]. Of note, ORF45 knockout KSHV has been used so far to study ORF45-mediated inhibition of IRF7 in infected cells. Additional KSHV mutagenesis studies could be employed to further dissect the impact of ORF45 on type I interferon production, as well as the general role of ORF45 in immune evasion in various cell types.

**Figure 3:**
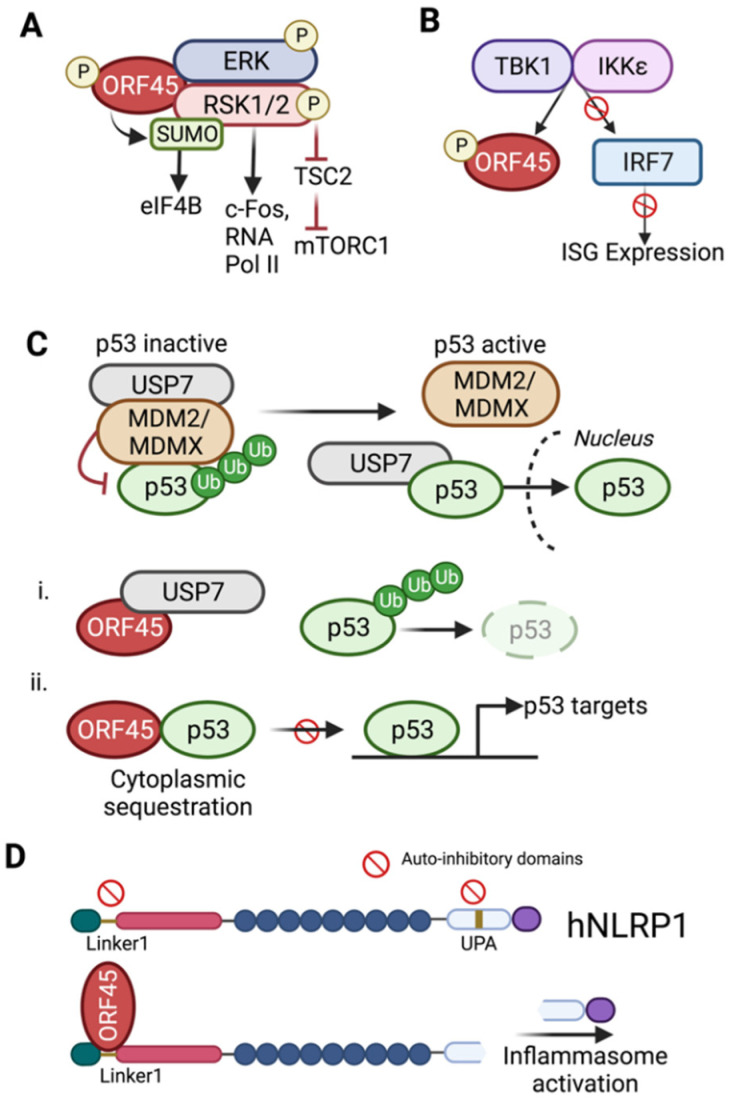
ORF45 interactions with host signaling pathways. (**A**) ORF45 sustains activation of the extracellular regulated kinase (ERK) p90 ribosomal s6 kinase (RSK) MAP kinase pathway by binding to the ERK/RSK complex and preventing their dephosphorylation. ORF45 also acts as a SUMO ligase and SUMOylates RSK to promote its kinase activity, which is essential for the phosphorylation of translation initiation complex factor eIF4B. RSK activity is also responsible for activation and nuclear accumulation of the c-Fos transcription factor, as well as recruitment of RNA polymerase II to promoters. RSK-mediated phosphorylation of the mTORC1 inhibitor, the tuberous sclerosis complex subunit TSC2, releases TSC2 inhibition to promote mTORC1 signaling during lytic infection. (**B**) ORF45 inhibits interferon regulator factor 7 (IRF7) activation and subsequent interferon stimulated gene expression by serving as an alternative phosphorylation substrate for upstream kinases TBK1 and IKKε (**C**) In the absence of external stimuli, p53 signaling is inhibited by E3 ubiquitin ligases MDM2/MDMX, which are stabilized by de-ubiquitinase USP7. Upon an appropriate external stimulus (e.g., DNA damage), p53 is released from MDM2/MDMX inhibition and stabilized by the binding of USP7, allowing p53 translocation to the nucleus and activation of downstream targets. ORF45 inhibits p53 signaling through (**i**) interaction and sequestration of p53 de-ubiquitinase USP7, which leads to p53 ubiquitylation and degradation and (**ii**) direct interaction and cytoplasmic sequestration of p53, which prevents p53 activation of its downstream transcriptional targets. (**D**) The hNLRP1 inflammasome is inhibited in steady state through interaction of auto-inhibitory domains in the Linker 1 region and the UPA component of the FIIND domain. ORF45 interaction with the Linker1 domain prevents this auto-inhibition leading to hNLRP1 C-terminal cleavage and inflammasome activation.

Furthermore, ORF45 has been recently characterized as an activator of the human NOD-like receptor-containing pyrin domain-1 (hNLRP1) inflammasome, which is accomplished through its disruption of NLRP1 auto-inhibition through binding to the Linker-1 region [46]. Inflammasome activation leads to the production of pro-inflammatory cytokines, which is also a characteristic feature of KS lesions, further highlighting an important role of ORF45 in fine-tuning the host immune response to viral infection in a highly context specific manner. Moreover, KSHV ORF45 expression was also sufficient to trigger inflammasome activation in cells transfected with rhesus or saimiri NLRP1, but not murine NLRP1, in which the Linker1 region is less conserved compared to the primate NLRP1, indicating a key evolutionarily conserved role for ORF45-mediated inflammasome activation in humans and non-human primates [46]. Further studies could evaluate the role of KSHV ORF45 homologs in NLRP1 binding and inflammasome activation. In sum, as highlighted in Figure 3, ORF45 has a broad-reaching effect on host signaling pathways.

## 10. ORF45 Homologs

The ORF45 protein is unique to the gammaherpesvirus family, with no homologs in alpha- or betaherpesviruses. ORF45 homologs in other gammaherpesviruses, including murine herpesvirus 68 (MHV68), Epstein Barr virus (EBV) and Rhesus monkey rhadinovirus (RRV) have been characterized, and are described below (Table 2).

### 10.1. MHV68 ORF45

Murine herpesvirus-68 (MHV68) is a murine virus related to KSHV and EBV. Studies of MHV68 can utilize the murine small animal, which is a powerful model system for gammaherpesvirus research. The ORF45 protein of MHV68 contains 206 amino acids and shares 33% sequence identity with KSHV ORF45 [83] and is present in both the cytoplasm and the nucleus following MHV68 infection [84]. The expression kinetics of MHV68 ORF45 differs from KSHV ORF45, as its expression is sensitive to cycloheximide treatment and slightly sensitive to phosphonoacetic acid treatment, inhibitors of protein synthesis and DNA replication, respectively, indicating that MHV68 ORF45 is an early-late protein and, unlike KSHV ORF45, MHV68 ORF45 requires viral protein translation in order to be expressed [85,86]. However, MHV68 is similar to KSHV ORF45 in that it is also part of the viral tegument [87,88], packaged into the virion in the outer tegument layer [89]. KSHV ORF45 has been shown to interact with another KSHV tegument protein, ORF33, which is important for the production of infectious virions [35,38]. Similarly, infection of cells with ORF33 knockout MHV68 leads to a deficiency of packaging of ORF45 into the mature virion, indicating a conserved interaction between tegument proteins ORF45 and ORF33 in MHV68 virion maturation [37].

Like KSHV ORF45, MHV68 ORF45 has a crucial role in the viral life cycle, and silencing ORF45 via RNA interference decreased viral protein expression and the production of viral progeny in infected cells [85]. Infection of baby hamster kidney (BHK)-21 cells with ORF45-knockout MHV68, which has a defect in both virion-associated ORF45 and newly synthesized ORF45, leads to a decrease in expression of late viral proteins and a decrease in DNA replication, which can be rescued by complementation with MHV68 ORF45 and partially rescued with KSHV ORF45 [90]. Similar to KSHV infection, the lack of newly synthesized ORF45 leads to a decrease in viral replication but not a decrease in late gene expression after one round of viral replication [84]. Specifically, the absence of MHV68 ORF45 affected virion maturation and envelopment, indicating that newly synthesized ORF45 is required for viral particle formation [84]. Furthermore, in contrast to KSHV ORF45, the role of MHV68 ORF45 in ERK/RSK activation is still largely unclear, which requires further investigation.

### 10.2. RRV ORF45

RRV is a nonhuman primate gammaherpesvirus that is closely related to KSHV and replicates to produce a high titer virus in vitro [91,92,93]. Analysis of RRV virion-associated proteins by mass spectrometry revealed that RRV ORF45 is also a putative tegument protein [94,95]. Like many DNA and RNA viruses, it has been shown that infection of rhesus fibroblasts with RRV leads to ERK activation [96]. Interestingly, while activated ERK2 is selectively packaged in the RRV virion tegument, knockdown of ERK1 was shown to promote viral infection, indicating distinct roles for ERK1 and ERK2 in the viral life cycle [96]. Like its KSHV homolog, expression of RRV ORF45 leads to sustained activation of the ERK/RSK pathway in rhesus fibroblasts and interacts with activated ERK2 and RSK to form a trimeric complex, which translocates to the nucleus [97]. The sustained activation of the ERK pathway may be partially responsible for the productive primary infections that are established following RRV de novo infections.

### 10.3. EBV BKRF4

Epstein Barr Virus (EBV) is a human oncovirus of the gammaherpesvirus family. EBV infections have been linked to multiple different cancers, such as Burkitt’s lymphoma, Hodgkin’s disease and nasopharyngeal carcinomas (Reviewed in [98,99]). The EBV protein BKRF4 is the homolog of KSHV ORF45, sharing a conserved region at the C-terminal amino acids, but otherwise with minimal sequence identity to KSHV ORF45 [100]. Antibodies towards BKRF4 have been detected in patients with nasopharyngeal carcinoma and have been suggested to have prognostic value [101]. Additionally, BKRF4 expression was detected in oral hairy leukoplakia lesions, an AIDS-associated lesion, which is also a biological site of replicating EBV [102]. Recently, BKRF4 was also identified in gastric carcinoma samples, indicating that it may be linked to oncogenesis [103]. However, the functional role of EBV BKRF4 has been less explored than its homolog in KSHV. BKRF4 has been identified as a tegument protein, similar to KSHV ORF45, but unlike KSHV ORF45 it is not an immediate early gene product. Instead, BKRF4 demonstrates early to late gene expression kinetics during lytic reactivation, with variable sensitivity to treatment with an inhibitor of viral DNA replication, phosphonoacetic acid [100,104].

While BKRF4 has been less studied as compared to KSHV ORF45, viral mutagenesis studies have revealed its role during lytic reactivation. Construction of a BKRF4 knockout virus revealed that the BKRF4-deficient virus had a comparable level of lytic viral gene expression, compared to wild-type virus following reactivation, but there was a clear reduction in viral progeny [100], a pattern which was also observed with ORF45 knockout KSHV [49]. Interestingly, the role of BKRF4 in infectious virion production is, in part, influenced by its C-terminal association with KSHV ORF33 homolog, BGLF2, a region which is similar to the ORF33 region known to interact with KSHV ORF45 [35,38,100]. BKRF4 localizes in the nuclear and perinuclear regions of cells but is excluded in a few small nuclear foci [100,105]. Additionally, BKRF4 has been shown to co-localize with other lytic proteins in the nucleus, including BGLF2 and BOLF1, a tegument protein and the homolog of KSHV ORF63 [106]. As a tegument protein, BKRF4 can play an immediate role in host immune evasion following primary infection. While BKRF4 has not been shown to inhibit IRF7, as has been shown for KSHV ORF45, the interference with the host DNA damage response suggests an alternative mechanism by which BKRF4 combats the host response. BKRF4 has been shown to inhibit the host DNA damage response by binding directly to histones and preventing histone ubiquitination at double-stranded DNA breaks by host ubiquitin ligase RNF168 [103]. In contrast to KSHV ORF45, which sustains activation of the RSK-signaling cascade, BKRF4 has not been shown to activate the MAP kinase pathway following infection. However, BKRF4 interacting partner, BGLF2, a homolog of KSHV ORF45, has been shown to play a role in activating the AP-1 family of transcription factors to promote EBV primary infection [107,108]. Given the limited conserved homology between BKRF4 and KSHV ORF45, further studies are required for identifying novel roles for tegument protein BKRF4 in EBV infection.

## 11. Conclusions

KSHV ORF45 plays a multifaceted but essential role in KSHV pathogenesis. As both a tegument protein and an immediate-early gene, ORF45 can contribute to both the initial phase of primary infections by promoting viral immune evasion, and also during the late stages of viral egress, by interacting with host motor proteins and cell membrane lipid rafts. Importantly, the ORF45-mediated sustained activation of the ERK/RSK pathway can lead to the activation of several host targets, many of which are likely to be highly context-specific and yet to be identified. While the contribution of KSHV ORF45 to virus production is well-studied, future research is needed to identify the cell type-specific functions of the ORF45 family of proteins encoded by gammaherpesviruses. Better understanding the role of ORF45 can also facilitate development of novel antiviral therapies. Additionally, the importance of KSHV ORF45 to the viral life cycle underscores that the contribution of tegument proteins cannot be understated. As the structure and function of the KSHV tegument continues to be unveiled, future work is needed to explore the key host-pathogen interactions facilitated by viral factors delivered directly into the host cell during infections, which are also capable of rapid global host reprogramming.

## Figures and Tables

**Table 1:** Previously characterized ORF45 mutants/motif.

ORF45 Mutant/Motif	Function	Reference
S41A/S162A	IKKε and TBK1 phosphorylation sites	[41]
F66A	ERK/RSK binding and activation	[42]
A144G/V146G	SIAH-1 binding site	[34]
E223A(G224E)/S226A	USP7 binding site	[35,43]
_237_IVDL_240_/_328_VIII_331_ mutant	SIM1 and SIM2 binding	[44]
V284A/L285A; I289A/L291A	ORF45 restricted to the nucleus (NES mutant)	[23]
K297R	ORF45 restricted to the cytoplasm (NLS mutant)	[23]
K297R, K99R, (297–300)4A	Targeting capsid to lipid rafts	[45]
Δ300-332	hNLRP1 binding site	[46]
W403A/W405A	ORF33 binding site	[38]

**Table 2:** Summary of ORF45 homologs.

Protein	Length (aa)	Expression Kinetics	Conserved Motif	Known Functions
KSHV ORF45	407	Tegument, immediate-early	N terminus C terminus	ERK/RSK activation, ORF33 binding, production of viral progeny, IRF7 inhibition, inflammasome activation, SUMO E3 ligase
MHV 68 ORF45	217	Tegument, early/late	N terminus C terminus	ORF33 binding, production of viral progeny
RRV ORF45	353	Tegument, early	N terminus C terminus	ERK/RSK activation, ORF33 binding, production of viral progeny, SUMO E3 ligase
EBV BKRF4	206	Tegument, early/late	N terminus C terminus	BGLF2 (ORF33) binding, production of viral progeny, inhibition of host DNA damage response

## Data Availability

Not applicable.
